# Revealing the frequency-dependent oscillations in the nonlinear terahertz response induced by the Josephson current

**DOI:** 10.1093/nsr/nwad163

**Published:** 2023-06-01

**Authors:** Sijie Zhang, Zhiyuan Sun, Qiaomei Liu, Zixiao Wang, Qiong Wu, Li Yue, Shuxiang Xu, Tianchen Hu, Rongsheng Li, Xinyu Zhou, Jiayu Yuan, Genda Gu, Tao Dong, Nanlin Wang

**Affiliations:** International Center for Quantum Materials, School of Physics, Peking University, Beijing 100871, China; State Key Laboratory of Low-Dimensional Quantum Physics and Department of Physics, Tsinghua University, Beijing 100084, China; International Center for Quantum Materials, School of Physics, Peking University, Beijing 100871, China; International Center for Quantum Materials, School of Physics, Peking University, Beijing 100871, China; International Center for Quantum Materials, School of Physics, Peking University, Beijing 100871, China; International Center for Quantum Materials, School of Physics, Peking University, Beijing 100871, China; International Center for Quantum Materials, School of Physics, Peking University, Beijing 100871, China; International Center for Quantum Materials, School of Physics, Peking University, Beijing 100871, China; International Center for Quantum Materials, School of Physics, Peking University, Beijing 100871, China; International Center for Quantum Materials, School of Physics, Peking University, Beijing 100871, China; International Center for Quantum Materials, School of Physics, Peking University, Beijing 100871, China; Condensed Matter Physics and Materials Science Department, Brookhaven National Lab, Upton, NY 11973, USA; International Center for Quantum Materials, School of Physics, Peking University, Beijing 100871, China; International Center for Quantum Materials, School of Physics, Peking University, Beijing 100871, China; Beijing Academy of Quantum Information Sciences, Beijing 100913, China

**Keywords:** high-temperature superconducting cuprates, nonlinear optics, time-domain tarahertz spectroscopy

## Abstract

Nonlinear responses of superconductors to intense terahertz radiation has been an active research frontier. Using terahertz pump-terahertz probe spectroscopy, we investigate the *c*-axis nonlinear optical response of a high-temperature superconducting cuprate. After excitation by a single-cycle terahertz pump pulse, the reflectivity of the probe pulse oscillates as the pump-probe delay is varied. Interestingly, the oscillatory central frequency scales linearly with the probe frequency, a fact widely overlooked in pump-probe experiments. By theoretically solving the nonlinear optical reflection problem on the interface, we show that our observation is well explained by the Josephson-type third-order nonlinear electrodynamics, together with the emission coefficient from inside the material into free space. The latter results in a strong enhancement of the emitted signal whose physical frequency is around the Josephson plasma edge. Our result offers a benchmark for and new insights into strong-field terahertz spectroscopy of related quantum materials.

## Teaser

The intense terahertz-induced nonlinear responses of Josephson current along the c-axis of high-temperature superconducting cuprates are investigated both experimentally and theoretically.

## INTRODUCTION

The electrodynamic responses of high-temperature superconducting cuprates (HTSCs) are highly anisotropic. Conducting carriers are substantially constrained within the two-dimensional CuO_2_ layers, while coherent out-of-plane (*c*-axis) charge transport is not allowed in the normal state [1]. Below the transition temperature T_*c*_, neighboring CuO_2_ layers are coupled by the Josephson tunneling of Cooper pairs [2–5]. At the linear response level, the *c*-axis optical conductivity has a Drude form with a zero scattering rate and a plasma frequency being the Josephson plasmon mode (JPM) frequency ω_JPR_. This means that weak electromagnetic fields with frequency below ω_JPR_ are forbidden to propagate due to the screening of Josephson currents, while those above ω_JPR_ are admissive, leading to a sharp Josephson plasmon edge (JPE) near ω_JPR_ in the *c*-axis reflectance spectrum [6]. The appearance of the JPE implies the formation of three-dimensional superconductivity and the emergence of the JPM along the *c* axis.

In the nonlinear regime, the nonlinear responses induced by Josephson tunneling may also be cast in the form of odd-order nonlinear susceptibilities (see the online supplementary material). Since the JPE of HTSCs usually lies in the terahertz (THz) range, the nonlinear response induced by intense THz radiations is anticipated to exhibit special features. Thanks to the advent of femtosecond laser techniques, strong-field THz pulse with stable carrier-envelope phase (CEP) has been a powerful tool in detecting and manipulating exotic quantum states, by introducing nonlinear processes without injecting overwhelmed energy [7–15]. Recently, several nonlinear electromagnetic responses related to the JPM using CEP-stable strong-field THz pulses have been reported [16–19]. Rajasekaran *et al.* [16] found that, in the THz pump-THz probe measurement along the *c* axis of superconducting La_1.905_Ba_0.095_CuO_4_, temporal oscillations centered at 2ω_JPR_ appear in the pump-probe delay process after the excitation of single-cycle THz pulses. Subsequently, they also observed a giant third-harmonic generation of single-cycle broadband THz pulses [17].

In this paper, we perform THz pump-THz probe spectroscopy on La_1.905_Ba_0.095_CuO_4_ and investigate the out-of-plane transient optical responses after the excitations of single-cycle THz pulses and multi-cycle ones. We observe long-lasting temporal oscillatory signals in the pump-probe decay process of the superconducting state after THz excitations. Surprisingly, by discerning the oscillatory behavior at different THz probe frequencies, we find that the frequency of oscillations induced by the single-cycle THz pump depends linearly on the probe frequency. In contrast, the multi-cycle THz pump centered at ω_pump_ induces single-frequency oscillations near 2ω_pump_, whose amplitude reaches a maximum as ω_pump_ approaches ω_JPR_. By plotting the nonlinear signal on the two-dimensional plane of the pump and probe frequencies, we reveal the origin of these behaviors as an enhancement when the physical frequency of the signal is around ω_JPR_. This is explained simply by the emission coefficient that we derive from solving the nonlinear electromagnetic (EM) problem for the interface, while the resonant excitation of a single collective mode is not assumed. Our result shows that in pump-probe nonlinear spectroscopy, this emission coefficient needs to be included in order to extract meaningful information about the probed material.

## RESULTS

**Figure 1.**
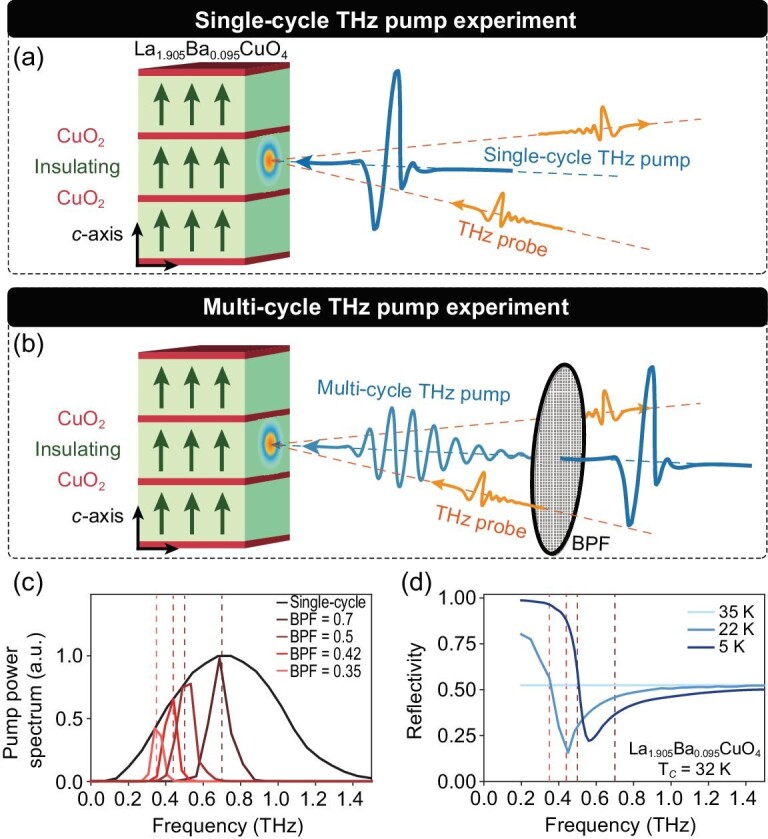
Experimental setup and sample characterization. (a and b) Schematics of the THz pump-THz probe spectrometers with broad- and narrow-band THz pump. Both the THz pump and probe are paralleled with the *c* axis of La_1.905_Ba_0.095_CuO_4_. Green arrows indicate the direction of the Josephson tunneling current. BPF represents a band-pass filter. (c) Power spectra of the broadband THz pump (black) and narrow-band ones with center frequencies of 0.35, 0.42, 0.5 and 0.7 THz (colored). (d) The *c*-axis reflectance spectra where sharp Josephson plasmon edges show up at T < T_*c*_.

Figure 1 summarizes the configurations of the THz pump-THz probe experiments. The schematic setups for single- and multi-cycle THz pump experiments are illustrated in panels (a) and (b) of Fig. 1. The single-cycle THz pump is centered at 0.7 THz with a peak electric field of ∼200 kV/cm, shown as the black curve in Fig. 1(c). The multi-cycle THz pump pulses are centered at 0.35, 0.42, 0.5 and 0.7, with peak electric fields of 30, 40, 60 and 70 kV/cm, respectively. The polarization of the THz pump and probe is set to be parallel to the *c* axis of the HTSC sample. For the single-layer HTSC La_2−*x*_Ba_*x*_CuO_4_, there is only a uniform Josephson coupling strength along the *c* axis. In addition, the stripe order within CuO_2_ layers, which can restrain the Josephson tunneling and compete with superconductivity, is nearly absent when the doping level is away from *x* = 0.125 [20]. To avoid the complicated interactions between different JPMs and those between the JPM and stripe order, we measured the sample without a stripe order phase, La_1.905_Ba_0.095_CuO_4_, whose T_*c*_ is 32 K. The *c*-axis reflectance spectra in the THz regime are presented in Fig. 1(d). In the normal state, an insulating-like response is observed. Having entered the superconducting state, a sharp JPE shows up. As the temperature decreases, the JPE shifts to a higher energy due to the increase in the superfluid density.

**Figure 2.**
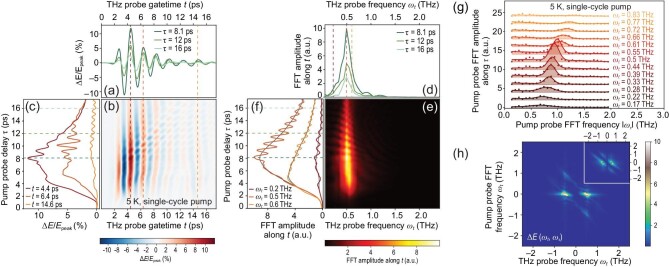
Single-cycle THz pump experiment at 5 K. (a) The pump-induced changes along electro-optic sampling gate time *t* of the reflected THz probe pulse at certain pump-probe delay times τ, Δ*E*(*t*, τ). (b) A two-dimensional time-domain Δ*E*(*t*, τ) color plot. (c) The decay profiles of Δ*E*(*t*, τ) at three gate times, in which clear oscillations are observed. The oscillation period approaches 1 ps as *t* increases from 4.4 to 14.6 ps. (d) The pump-induced changes in the frequency domain ω_*t*_, Δ*E*(ω_*t*_, τ). (e) The two-dimensional color plot of Δ*E*(ω_*t*_, τ) presents a temporal modulation in a series of backward-slashes patterns along τ. (f) The decay profiles of Δ*E*(ω_*t*_, τ) at three representative ω_*t*_. The oscillating signals can be obtained by subtracting away the nonoscillating components plotted as the thin black curves. (g) FFT amplitude of the extracted oscillatory signals in Δ*E*(ω_*t*_, τ) is plotted as scatters and fitted with solid curves. (h) The two-dimensional FFT of the measured Δ*E*(*t*, τ) shown in (b), Δ*E*(ω_*t*_, ω_τ_). Inset: Δ*E*(ω_*t*_, ω_τ_) estimated by numerically solving the third-order nonlinear responses.

Figure 2 summarizes the out-of-plane transient responses at 5 K after the excitation by single-cycle THz pulses. The raw data are the pump-induced changes of the reflected THz probe scanned along the electro-optic sampling gate time *t* (defined as the time difference between the gate time and the arrival time of the probe pulse) at a certain pump-probe delay time τ (the time difference between the gate time and the arrival time of the pump pulse), i.e. Δ*E*(*t*, τ) shown in Fig. 2(a). A two-dimensional time-domain color plot of Δ*E*(*t*, τ) shown in Fig. 2(b) is achieved by scanning over τ. The decay profiles of Δ*E*(*t*, τ) at representative *t* shown in Fig. 2(c) are obtained by cutting along the τ axis. To further clarify the transient responses, the pump-induced changes in the frequency domain are studied. By performing the fast Fourier transformation (FFT) on Δ*E*(*t*, τ) along the *t* axis, Δ*E*(ω_*t*_, τ) is determined, as shown in Fig. 2(d). The pump-induced change primarily happens near ω_JPR_ ∼ 0.5 THz. The corresponding transient optical properties show a splitting of the JPM induced by the THz pump, as presented in the online supplementary material, similar to the responses after mid- and near-infrared excitations [21,22]. Figure 2(e) shows the two-dimensional color plot of Δ*E*(ω_*t*_, τ), which is temporally modulated by a series of backward-slash patterns along τ. The slopes of those slashes are different, which suggests that the τ-axis oscillation period is not a constant along the THz probe frequency ω_*t*_. Figure 2(f) shows the pump-probe decay profiles at three representative ω_*t*_.

Those oscillatory signals along τ are extracted by subtracting away the nonoscillating pump-probe curves (thin black curves in Fig. 2(f)). The oscillatory amplitude is shown in Fig. 2(g). There is a clear blueshift of the oscillatory center frequency as the THz probe frequency ω_*t*_ increases, which indicates a linear relationship between ω_τ_ and ω_*t*_. Identical oscillatory behaviors also exist in the transient optical constants, as presented in the online supplementary material. Although temporal oscillations in pump-probe spectral experiments have been widely observed, those oscillations usually center at a fixed frequency and correspond to an excitation of some specific collective mode [23] such as coherent phonons, amplitude modes in charge density wave compounds and Higgs modes. The linear varying behavior reported here has not been reported before. For the THz-induced transient out-of-plane responses of La_1.905_Ba_0.095_CuO_4_ reported in [16], the oscillation along the pump-probe delay τ centers at the fixed frequency 2ω_JPR_ since they were looking at a fixed *t*, which may be viewed as a special case of the linear-dependent relation reported here.

Figure 2(h) shows the two-dimensional FFT of the measured Δ*E*(*t*, τ) shown in Fig. 2(b), Δ*E*(ω_*t*_, ω_τ_). There are four bright spots in Fig. 2(h), which are located at (±0.5, 0), (0.5, −1) and (−0.5, 1). The bright spots at (±0.5, 0) correspond to the nonoscillating component along the τ axis in Fig. 2(f), and the ones at (0.5, −1) and (−0.5, 1) correspond to the oscillating component. It is worth noting that the bright spots at (0.5, −1) and (−0.5, 1) are stretched along the direction ω_*t*_ + ω_τ_ = ±0.5, which reflects the linear relationship between ω_τ_ and ω_*t*_ observed in Fig. 2(g).

We now try to interpret that linear dependence by THz nonlinear electrodynamics. Since there is inversion symmetry in La_1.905_Ba_0.095_CuO_4_, the leading nonlinear optical process must be due to the third-order nonlinear optical response. Indeed, the measured signal scales linearly with the probe field and quadratically with the pump field. By solving the full EM problem, we derive the pump-induced change of the reflected probe field Δ*E*(*t*, τ) as


(1) \begin{eqnarray*}
\Delta E(t,\tau )&=& \sum _{\omega _1, \omega _2, \omega _3}F(\omega , \mathbf {k})\chi ^{(3)}{(\omega _1, \omega _2,\omega _3)} \\
&&\cdot E_{\text{pump}}(\omega _1)E_{\text{pump}}(\omega _2)E_{\text{probe}}(\omega _3) \\
&&\cdot e^{-i(\omega _1+\omega _2) \tau -i \omega _3 t},
\end{eqnarray*}

where *E*_pump_ and *E*_probe_ are the electric fields of transmitted pump and probe pulses inside the sample, χ^(3)^ is the third-order nonlinear susceptibility defined as the ratio of the third-order polarization to the incident fields, *F* is the emission coefficient of the third-order polarization into the far field, and (ω = ω_1_ + ω_2_ + ω_3_, **k**) is the frequency and momentum of the polarization generated from the third-order nonlinear effect (see Section 3 of the online supplementary material for the derivation). The Josephson relation predicts that


\begin{eqnarray*}
\chi ^{(3)}(\omega _1, \omega _2, \omega _3) \!=\! \frac{(2e)^3}{3!} \frac{J_c d^3}{(\omega _1\!+\! \omega _2\!+\! \omega _3)\omega _1 \omega _2 \omega _3},
\end{eqnarray*}

where *J_c_* is the Josephson critical current density, *d* is the inter-CuO_2_ layer distance and *e* is the elementary charge. The Fourier transform of Δ*E*(*t*, τ) is therefore


(2) \begin{eqnarray*}
\Delta E (\omega _t,\omega _\tau )&=&\sum _{\omega } F(\omega _t+\omega _\tau ,\mathbf {k}) \chi ^{(3)} \\
&&\cdot(\omega ,\omega _\tau -\omega , \omega _t) E_{\text{pump}}(\omega ) \\
&&\cdot E_{\text{pump}}(\omega _\tau -\omega ) E_{\text{probe}}(\omega _t).\\
\end{eqnarray*}

Since the signal is a product of two pumps and one probe, its frequency is equal to the sum of the frequency of each constitute. Thus, Δ*E*(ω_*t*_, ω_τ_) in Equation (2) means the amplitude of the third-order signal with physical frequency ω_physical_ = ω_τ_ + ω_*t*_. The incident pump/probe pulses are modeled as Gaussian functions reshaped by the transmission coefficient *T*(ω):


(3a) \begin{eqnarray*}
E_{\text{probe}}(\omega ) &=&T(\omega )E_{\text{pr}} (e^{-(\omega -\omega _{\text{pr}})^2/W^2_{\text{pr}}}\\
&& +\ e^{-(\omega +\omega _{\text{pr}})^2/W^2_{\text{pr}}} ),
\end{eqnarray*}

(3b) \begin{eqnarray*}
E_{\text{pump}}(\omega )& =& T(\omega ) E_{\text{pu}} (e^{-(\omega -\omega _{\text{{pu }}})^2/W^2_{\text{pu}}}\\
&&+\ e^{-(\omega +\omega _{\text{{pu }}})^2/W^2_{\text{pu}}} ).
\end{eqnarray*}

Here the central frequencies ω_pu_/ω_pr_ and spectrum widths *W*_pu_/*W*_pr_ are determined by measurements. Note that *T*(ω) is calculated from the optical conductivity inferred by the reflectance spectrum (Fig. 1(d)). Substituting Equations (3) into Equation (2) gives the spectrum of Δ*E*(ω_*t*_, ω_τ_).

The inset of Fig. 2(h) presents the numerical result of Equation (2), with ω_pu_ = 0.7 THz, ω_pr_ = 1 THz, *W*_pu_ = 0.6 THz and *W*_pr_ = 1 THz. The result explains all the bright spots in Fig. 2(h). Notably, the sharp bright lines along ω_*t*_ + ω_τ_ = ±ω_JPR_ may be explained by the sharp peaks in the emission coefficient *F*(ω, **k**) when the physical frequency ω = ω_*t*_ + ω_τ_ of the third-order nonlinear signal matches ±ω_JPR_, so that the wave vector |*k*_↓_|=|$\sqrt{\epsilon _c \omega ^2/c^2}$| of the emitted transverse EM mode inside the sample vanishes. The signal at this frequency emits most easily out of the sample. The same coefficient also suppresses the signals in the regions ω_*t*_, ω_τ_ > 0 and ω_*t*_, ω_τ_ < 0. Therefore, the above nonlinear response is well explained by the third-order nonlinear electrodynamics combined with the JPE. We remark that our approach goes beyond solving the dynamics of a nonlinear harmonic oscillator by considering the full EM problem on the interface between the material and space. Similar bright spots may occur in the two-dimensional spectroscopy of few-level systems [24], which merits further investigations.

**Figure 3.**
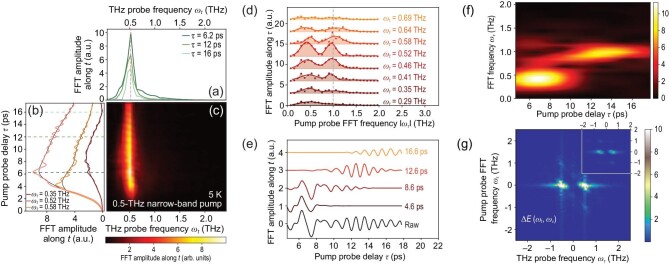
Multi-cycle THz pump experiment at 5 K. (a) The pump-induced changes in the frequency domain ω_*t*_, Δ*E*(ω_*t*_, τ), at three representative pump-probe delay times τ. (b) The decay profiles of Δ*E*(ω_*t*_, τ) at three ω_*t*_, in which oscillatory signals can be obtained by subtracting the nonoscillating background plotted as thin black curves. (c) The two-dimensional color plot of Δ*E*(ω_*t*_, τ), in which a temporal modulation is observed along τ. (d) FFT amplitude of the extracted oscillatory signals in Δ*E*(ω_*t*_, τ), which peaks near 0.5 and 1 THz. The gray dashed line is a visual guide. (e) Frequency-resolved optical gating technique is used for analyzing the time-varying oscillation period in (b), by applying a gating window to the raw oscillation at ω_*t*_ = 0.52 THz (black curve). By moving the gating window continuously, dominating signals near τ are extracted (colored curves). (f) FFT amplitude of the windowed oscillations. The oscillations near ω_pump_ dominate initial τ and long-lasting oscillations near 1 THz dominate the subsequent *t*. (g) The two-dimensional FFT of the measured Δ*E*(*t*, τ), Δ*E*(ω_*t*_, ω_τ_), in which four bright spots can be observed. Inset: Δ*E*(ω_*t*_, ω_τ_) estimated by numerically solving Equation (2).

We now investigate the out-of-plane transient responses to CEP-stable multi-cycle THz pulses, which has not been explored before. Figure 3 presents the experimental results after 0.5-THz multi-cycle THz excitations. Here Δ*E*(ω_*t*_, τ) also peaks around ω_JPR_, with a temporal modulation along τ, as shown in panels (a) and (b) of Fig. 3. Figure 3(c) shows the two-dimensional color plots of Δ*E*(ω_*t*_, τ), in which nearly no ω_*t*_-dependent modulation is observed. Figure 3(d) shows the oscillation amplitude at different ω_*t*_, which all peak around 0.5 and 1 THz. We note that those pump-induced oscillations are invisible after excitations of CEP-unstable narrow-band THz pulses radiated from a free-electron laser [25]. Furthermore, the oscillation frequencies are nearly independent of the THz probe frequency ω_*t*_. We also note that the oscillation period in Fig. 3(b) varies in time, which can be analyzed by a frequency-resolved optical gating technique [26]. As shown in Fig. 3(e), gating windows in a Gaussian form with a duration of 3 ps are applied to the extracted raw oscillatory signals. By moving the gating window continuously in the pump-probe delay, dominant signals near τ are extracted, of which the FFT amplitudes are presented in Fig. 3(f). That time-frequency distribution analysis shows that the quasi-single-cycle oscillation near 0.5 THz only exists during the initial τ, while long-lasting oscillation near 1 THz dominates the subsequent τ. The oscillation at ω_pump_ = 0.5 THz may indicate a broken inversion symmetry, which warrants further investigation. The long-lasting oscillation near 1 THz could be either 2ω_pump_ or 2ω_JPR_, which will be further investigated by pump-wavelength- and temperature-dependent experiments below. Figure 3(g) shows the two-dimensional Δ*E*(ω_*t*_, ω_τ_). The bright spots at (0.5, −1) and (−0.5, 1) are nearly round and not stretched, which indicates that the oscillatory central frequency along the τ axis is almost a constant, as shown in Fig. 3(d). The inset of Fig. 3(g) shows the calculation result of Equation (2), with ω_pu_ = 0.5 THz, ω_pr_ = 1 THz, *W*_pu_ = 0.1 THz and *W*_pr_ = 1 THz, whose pattern is qualitatively the same as Fig. 3(g). According to Equation (2), the limited bandwidth of pump pulses results in the constant oscillatory central frequency.

**Figure 4.**
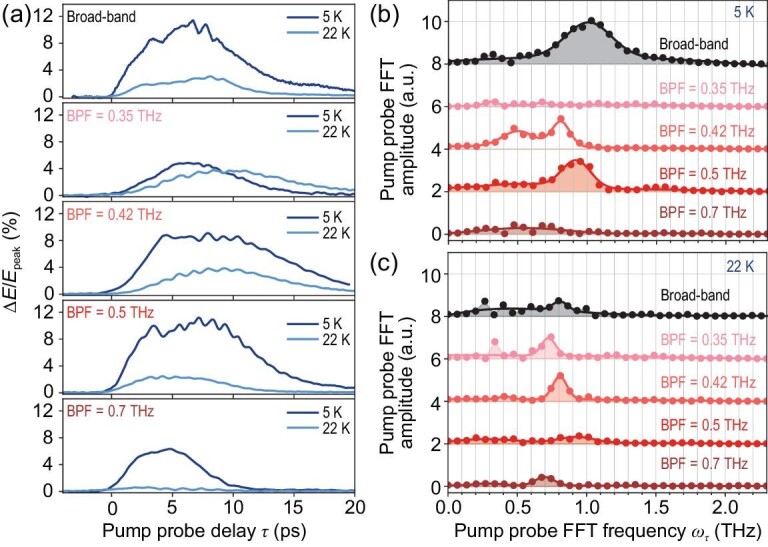
Pump-wavelength- and temperature-dependent experiments. (a) The pump-probe decay profiles of Δ*E*(*t* = 4.2 ps, τ) after different excitations at 5 and 22 K. FFT amplitudes of the extracted oscillatory signals in Δ*E*(*t* = 4.2 ps, τ) at (b) 5 K and (c) 22 K.

Figure 4(a) shows the pump-probe decay profiles of Δ*E*(*t* = 4.2 ps, τ) at 5 and 22 K after the excitations of different THz pumps, which is obtained by fixing *t* to 4.2 ps and scanning over τ. In panels (b) and (c) of Fig. 4 we present the amplitudes of oscillatory signals extracted from Fig. 4(a). For comparison, a temperature-dependent measurement was first performed for the single-cycle THz pump case, in which the oscillation central frequency shifts from 1 to 0.8 THz due to a redshift of the JPM as the temperature increases from 5 to 22 K.

For multi-cycle THz pumps centered at 0.35 and 0.7 THz, which are significantly off-resonant with ω_JPR_ at 5 K, nearly no oscillatory signals can be recognized at 5 K. Upon tuning ω_pump_ to 0.42 THz, weak oscillations centered at 0.8 THz show up. For the 0.5-THz pump resonant with ω_JPR_ at 5 K, the oscillations are maximized, with a central frequency of 1 THz. The pump-wavelength-dependent experiment confirms that the long-lasting oscillations induced by a multi-cycle THz pump do peak around 2ω_pump_. Upon increasing the temperature to 22 K, the JPM shifts to ∼0.4 THz and becomes more damped. The oscillation amplitude at 22 K reaches a maximum when ω_pump_ = 0.42 THz. For the 0.35-THz pump, clear oscillations at 0.7 THz emerge, quite different from the nonoscillating behaviour at 5 K. For the 0.5-THz pump, oscillations still peak at 1 THz but become almost invisible. As the temperature approaches T_*c*_, the JPM moves out of the measurement range and nearly no pump-induced changes are detectable. To summarize, for the multi-cycle THz pump centered at ω_pump_, the central frequency of τ-axis long-lasting oscillations is equal to 2ω_pump_. The oscillation amplitude is maximized when ω_pump_ resonates with ω_JPR_, which could result from the filtering effect imposed by JPE and the emission coefficient *F*(ω, **k**).

We now discuss the origin of the long-lasting (∼20 ps) temporal oscillations in the pump-probe process that is also revealed by the sharp lines in Fig. 2(h) with widths ∼0.1 THz. We proposed that the oscillations were due to the sharp peaks in the emission coefficient *F* in Equation (1). Another possible reason is the transmission peak (Fig. 1(d)) of the interface around the JPM that may lead to a narrow frequency signal inside the sample even for a broadband single-cycle pump. However, the width of the transmission peak is far too broad to explain it.

Alternatively, the oscillations may come from excitation of a long-lived collective mode around ω_JPR_. Of course, the EM waves inside the sample excited by the pump and probe may be called (hyperbolic) Josephson plasmons[27], but they span a wide frequency range above ω_JPR_ instead of being a single mode (Section 3.4.1 within the online supplementary material). Inhomogeneity in the sample caused by disorder may introduce some longitudinal components to the incident field to excite the surface JPMs (Section 3.4.2 within the online supplementary material). However, if these effects existed, they should have added a Lorentzian oscillator around ω_JPR_ to the optical conductivity and corresponding features to the linear reflectivity [28], which are absent in Fig. 1(d). In the nonlinear pathway, JPMs with opposite momenta may be parametrically excited in pairs by the pump [29–31]. Again, since the JPMs have a wide dispersion covering a large frequency range, this process would result in continuous exciting spectra instead of a well-defined resonance around ω_JPR_. Moreover, being proportional to quantum fluctuations of the JPM, the plasmon-pair contribution [29] is much weaker than the tree-level nonlinearity considered here (Section 3.3 within the online supplementary material). The relevance of this interesting contribution [29] in our experiment requires future investigation. Finally, the Higgs amplitude mode has been excited nonlinearly before using strong THz radiation in conventional superconductors [26,32–36] and within the CuO_2_ layers of HTSCs [37–39]. Unfortunately, the Higgs mode is irrelevant here since its frequency of ∼1.7Δ_0_ [40] is much higher than those of the THz pump pulses in our experiments where 2Δ_0_ = 30 meV is the gap at the anti-nodal position [41]. This conclusion is further reinforced by the multi-cycle THz pump experiment, which demonstrates a resonance in the oscillation when ω_pump_ approaches ω_JPR_.

In conclusion, in the THz pump-THz probe experiments performed on La_1.905_Ba_0.095_CuO_4_, long-lasting temporal oscillations are observed in the out-of-plane transient responses of the superconducting state during the pump-probe decay process, which results from the *c*-axis third-order nonlinear response together with interesting frequency dependence of the emission coefficient. For the single-cycle THz pump, the oscillation frequency is linearly dependent on the frequency of detection of the THz probe beam and is also closely related to the frequency of the JPE, rather than centered at a fixed frequency. In contrast, the multi-cycle THz pump centered at ω_pump_ leads to the most dramatic oscillations once ω_pump_ approaches ω_JPR_. We conclude that the observed oscillations in the pump-probe delay probably come from the sharp emission peak close to the JPM, an EM phenomenon, while the resonant excitation of a single collective mode is not necessary to explain it. The result of this study not only provides a benchmark of nonlinear optical responses of HTSCs, but also offers new insight into the emerging frontier of strong-field THz spectroscopy on related complex quantum materials. In the future, with Equation (2) applied to single color THz pump-probe spectroscopy, it will be possible to map out the nonlinear susceptibility quantitatively, offering more direct information of the material.

## METHODS

The strong-field THz pump with stable carrier-envelope phase is generated using the tilted pulse-front method. The THz probe is generated and detected by ZnTe crystals. The polarization of the THz pump and probe is set to be parallel to the *c* axis of the crystal. The detailed schematic optical path diagram can be found in the online supplementary material. The single crystal is grown using the traveling-solvent floating-zone method. The *c* axis is acquired by cutting the a-b surface at 90° with a subsequent polishing.

## Supplementary Material

nwad163_Supplemental_File Supplementary data are available at *NSR* online. which include [42–49].
