# Supplementary material for: Revealing the frequency-dependent oscillations in the nonlinear terahertz response induced by the Josephson current
Source: Natl Sci Rev. 2023 Jun 1;10(11):nwad163. doi: 10.1093/nsr/nwad163 (PMC10561709; doi:10.1093/nsr/nwad163)
Supplement: nwad163_Supplemental_File — Supplementary data are available at NSR online. which include [42–49]. [file nwad163_supplemental_file.pdf]

Revealing the frequency-dependent oscillations in nonlinear terahertz response induced by Josephson current

S. J. Zhang[1,*], Z. Sun[2,*], Q. M. Liu[1], Z. X. Wang[1], Q. Wu[1], L. Yue[1], S. X. Xu[1], T. C.Hu[1], R. S. Li[1], X. Y. Zhou[1], J. Y. Yuan[1], G. D. Gu[3], T. Dong[1], and N. L. Wang[1,4,*]

[1]International Center for Quantum Materials, School of Physics, Peking University, Beijing 100871, China
[2]State Key Laboratory of Low-Dimensional Quantum Physics and Department of Physics, Tsinghua University, Beijing 100084, China
[3]Condensed Matter Physics and Materials Science Department,Brookhaven National Lab, Upton, New York 11973, USA
[4]Collaborative Innovation Center of Quantum Matter, Beijing, China

Supplementary Information

1 Schematic Optical Path Diagram

Figure S1 shows the schematic optical path diagram of the terahertz (THz) pump-THz probe spectrometer in reflection geometry. The spectrometer is based on a regenerative amplified Ti: sapphire laser system with 800-nm center wavelength, 4-mJ pulse energy, ~100-fs pulse duration, and 1-kHz repetition rate. The laser output was divided into three beams: for the generation of the pump and probe THz pulses, and the gate pulse for electro-optic sampling (EOS) the reflected THz probe, as shown in the lower panel of Fig. S1. EOS gatetime t is the relative delay between probe and EOS gate pulses, and pump-probe delay time τ is the relative delay between pump and EOS gate pulses.

There are two optical delay lines in the spectrometer, which are set along the THz probe path (Delay line I) and EOS gate one (Delay line II), respectively. Delay line I is used for controlling the EOS gatetime t and Delay line II for pump-probe delay τ. By moving Delay line I while keeping Delay line II at a specific τ, we can get the pump-induced changes of the reflected THz probe pulse along EOS gatetime t at certain pump-probe delay time τ, *i.e.* $\Delta E(t, \tau)$ shown in Fig. 2 (a) of the main text. A two-dimensional time-domain $\Delta E(t, \tau)$ scan shown in Fig. 2 (b) can be achieved by setting Delay line I and II at several τ simultaneously, and then scan $\Delta E(t, \tau)$. In addition, the decay profile of $\Delta E(t, \tau)$ at specific gatetime t shown in Fig. 4 (a) can be obtained by fixing the relative position of Delay line I and II at t, and then moving those two lines along τ simultaneously. Two choppers are put on the THz probe (Chopper I) and pump (Chopper II) beams, which are both triggered by the laser system and set to 500 and 250 Hz, respectively. Double modulation techniques are used for data acquisition[5], which can simultaneously obtain reflected THz probe without and after excitations.

The intense broad-band THz pump pulse was generated by the tilted pulse-front method on a $LiNbO_3$ crystal[4], and narrow-band THz pump ones are achieved by filtering the broad-band pulse with several metal-mesh band-pass filters. The time-domain EOS data of pump pulses are presented in Fig. S2, whose center frequency and bandwidths are plotted in Fig. 1 (e) of main text. The THz probe pulse is generated and detected by optical rectification and EOS on 1 mm-thick ZnTe crystals, which ranges from 0.2 to 2.5 THz. The THz pump pulse is focused into a ~ 2.2 mm-diameter spot near the sample position with a 90° off-axis parabolic mirror with focal length of 101.6 mm, while the THz probe one into a ~ 0.8 mm-diameter spot with a 30° off-axis parabolic mirror with focal length of 54.45 mm. Both the polarization of pump and probe pulses are set to be perpendicular to the optical table.

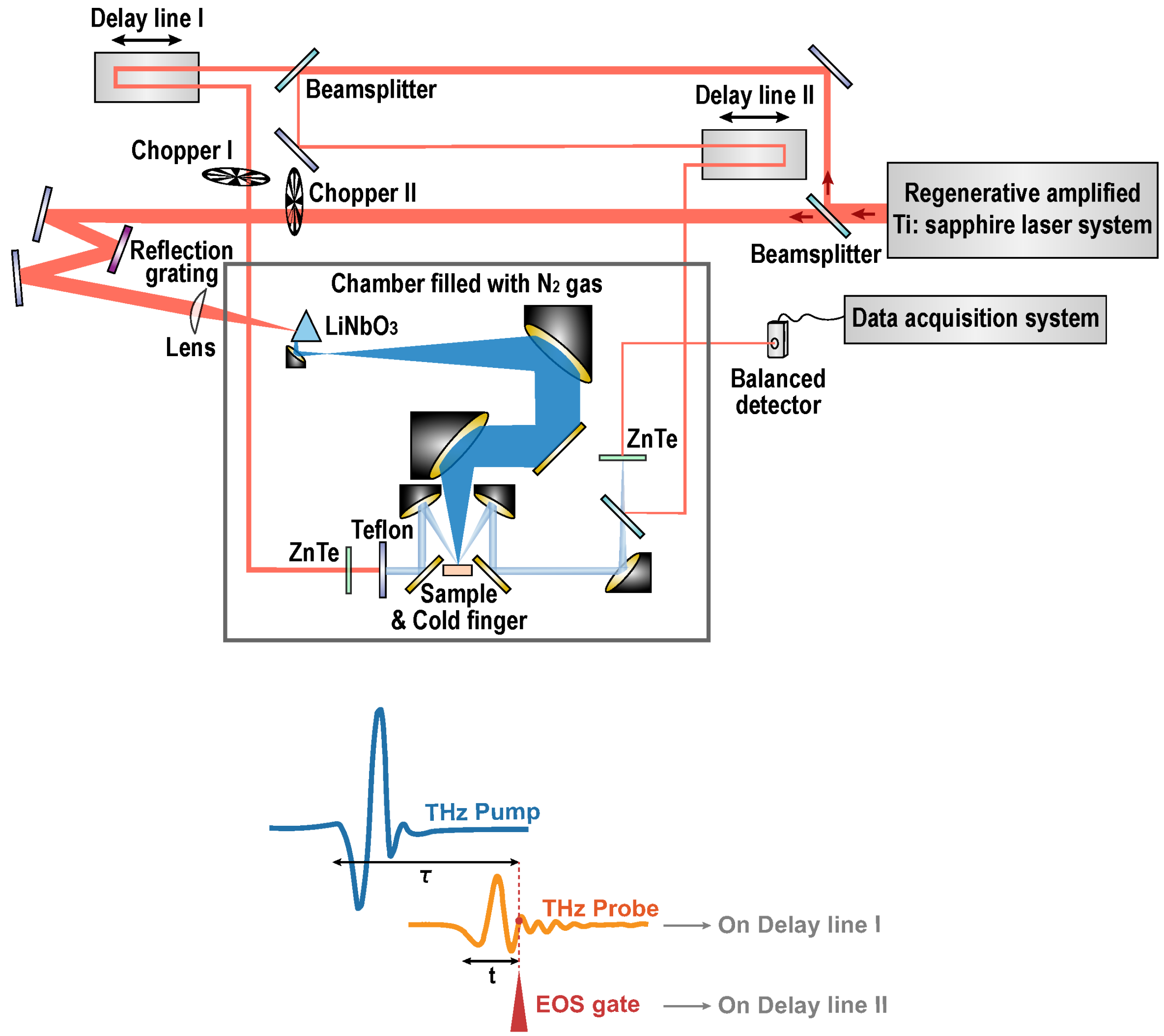

Figure S1: Upper panel: Schematic optical path diagram of THz pump-THz probe spectrometer in reflection geometry, in which some mirrors and lens are omitted for brevity. Lower panel: Schematic sketch of the relative delay time of optical pulses.

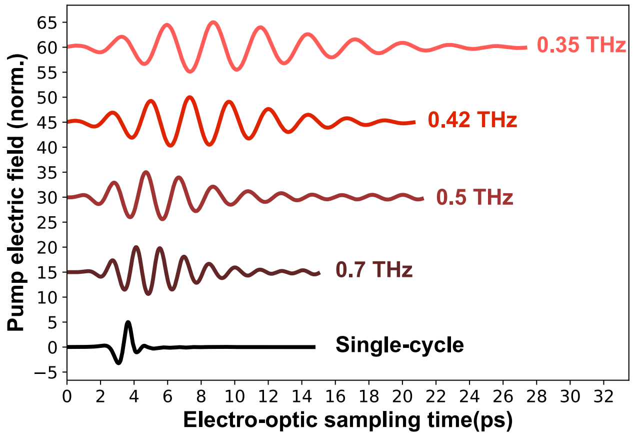

Figure S2: The time-domain electro-optic sampling data of pump pulses.

2 Transient Optical Properties after Terahertz Excitations

The complex optical constants in the equilibrium state can be precisely determined by a combination measurement of Fourier transform infrared spectroscopy and time-domain THz spectroscopy[9], which are the basis of acquiring the transient optical properties after excitations.

The raw measurement data of the THz pump-THz probe experiment are the electric fields of THz probe reflected from sample before and after excitations, *i.e.* $\tilde{E}_0(t)$ and $\tilde{E}'(t,\tau)$. After doing fast Fourier transformation, data in frequency domain $\tilde{E}_0(\omega_t)$ and $\tilde{E}'(\omega_t,\tau)$ are obtained, which simultaneously contain the amplitude and phase information. Then, we determine the transient complex reflected coefficient $\tilde{r}'(\omega_t,\tau)$ with

$$\tilde{r}'(\omega_t,\tau) = \frac{\tilde{E}'(\omega_t,\tau)}{\tilde{E}_0(\omega_t)} \cdot \tilde{r}_0(\omega_t), \tag{S1}$$

in which $\tilde{r}_0(\omega_t)$ is the complex reflected coefficient of the equilibrium state. Subsequently, the transient reflectivity $R(\omega_t,\tau)$ and complex refractive index $\tilde{N}(\omega_t,\tau)$ can be obtained with $R = |\tilde{r}|^2$ and Fresnel's Formula, respectively. All the other transient optical properties can be determined by $\tilde{N}(\omega_t,\tau)$.

Figure S3 shows the determined transient reflectivity $R(\omega_t,\tau)$ after single-cycle THz excitations at 5 K. The original sharp Josephson plasma edge at the equilibrium state is suppressed to lower energy scale while a new pump-induced edge shows up at higher energy scale, which has already been observed in the decay process after mid- and near-infrared excitations. Furthermore, clear temporal oscillatory signals are also observed in the pump-probe decay process of transient reflectivity. By extracting the oscillatory signals (Fig. S3 (d)) and then performing Fourier transformation (Fig. S3 (e)), we find the central frequency of oscillations in $R(\omega_t,\tau)$ (red scatters) is in coincidence with that in $\Delta E(\omega_t,\tau)$ (grey scatters), which is shown in Fig. 2 of the main text.

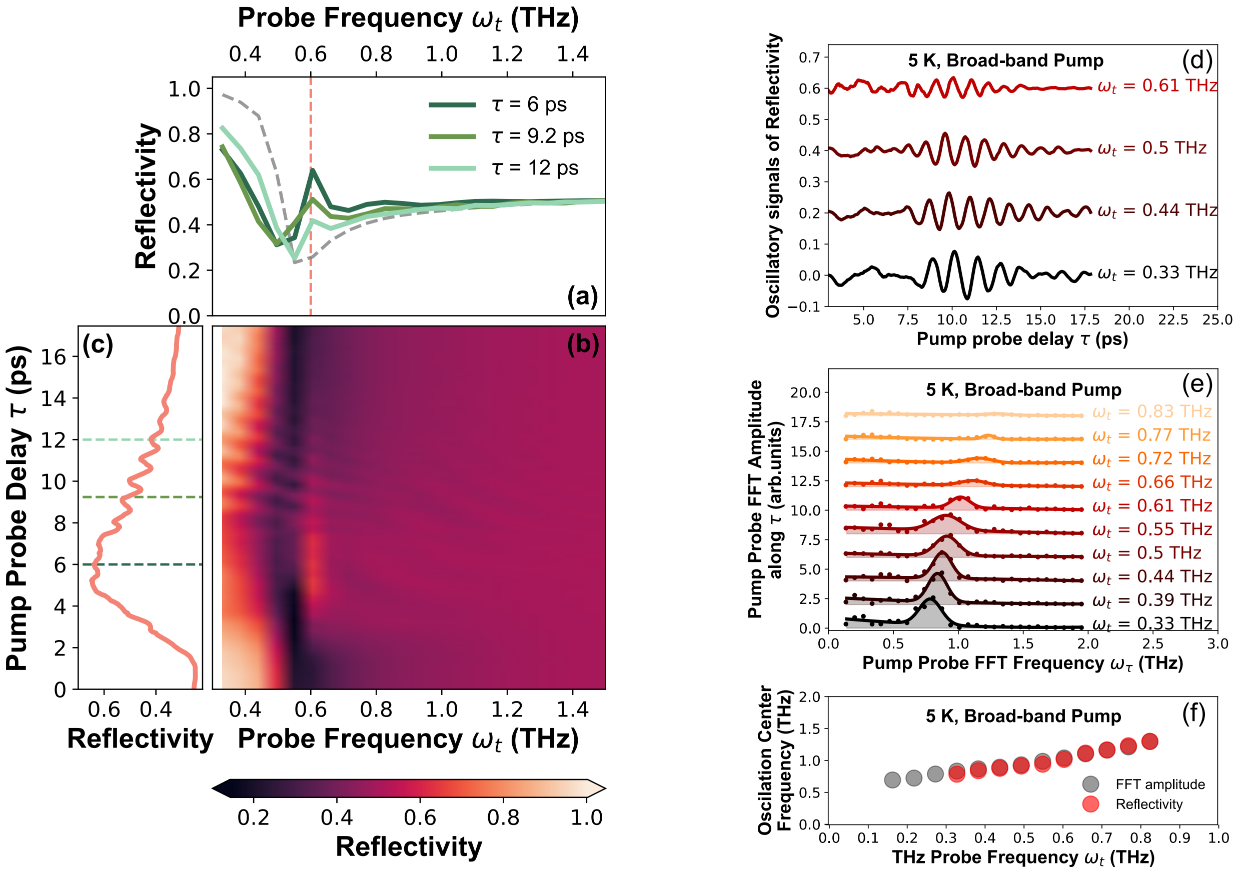

Figure S3: Transient reflectivity after single-cycle THz excitations at 5 K. (a) The transient reflectivity at certain pump-probe delay time τ, *i.e.* $R(\omega_t, \tau)$. (b) A two-dimensional color plot of $R(\omega_t, \tau)$. (c) The decay profiles of $R(\omega_t = 0.6\ \text{THz}, \tau)$. (d) The extracted oscillatory signals of transient reflectivity $R(\omega_t, \tau)$. (e) Fourier transformation of the extracted oscillatory signals are plotted as scatters and fitted with solid lines. (f) The center frequency ω_τ of the extracted oscillatory signals in $R(\omega_t, \tau)$ is plotted as red scatters, which is in coincidence with that in $\Delta E(\omega_t, \tau)$ (grey scatters) presented in the main text.

3 Theory of nonlinear optical reflection in layered superconductors

3.1 Results

The configuration is shown in Fig. S4. The pump and probe fields inside the material are related to the incident fields as $E_{\text{pump}} = TE_{\text{pump},0}$, $E_{\text{probe}} = TE_{\text{probe},0}$. We define $T(\omega, q)$ and $R(\omega, q)$ as the linear transmission and reflection coefficients at frequency ω and in-plane wave vector q. For TE polarization, their expressions are

$$R(\omega, q) = \frac{k_{\uparrow z} + k_{\downarrow z}}{k_{\uparrow z} - k_{\downarrow z}}, \quad T(\omega, q) = \frac{2k_{\uparrow z}}{k_{\uparrow z} - k_{\downarrow z}} \tag{S2}$$

where $k_{\uparrow z} = \sqrt{k_0^2 - q^2}$ and $k_{\downarrow z} = -\sqrt{\epsilon_c k_0^2 - q^2}$ are z-components of the wave vectors on the upper and lower side of the interface, and $k_0 = \omega/c$. Note that the branch of the complex square root needs to be chosen correctly such that both waves decay as they propagate away from the interface. The linear dielectric function along c-axis is

$$\epsilon_c(\omega) = \epsilon_{c\infty}\left(1 - \frac{\omega_{\text{JPR}}^2}{\omega^2}\right). \tag{S3}$$

To the second power in the pump field and first power of the probe field, the reflected third order signal is derived to be

$$E_{\uparrow}(\omega) = \sum_{\omega_i} F(\omega, \mathbf{k})\chi^{(3)}(\omega_1, \omega_2, \omega_3)E_{\text{pump}}(\omega_1, \mathbf{k}_1)E_{\text{pump}}(\omega_2, \mathbf{k}_2)E_{\text{probe}}(\omega_3, \mathbf{k}_3) \tag{S4}$$

where $F(\omega, \mathbf{k})$ is an emission coefficient that depends only on the frequency and momentum of the third order polarization induced by the pump and probe inside the sample. We will show its derivation in the following sections.

According to the definition of pump probe delay τ and the gate time t, the nonlinear signal can also be written for every ω_τ and ω_t:

$$\Delta E(\omega_t, \omega_\tau) = \sum_{\omega} F(\omega_\tau + \omega_t, \mathbf{k})\chi^{(3)}(\omega, \omega_\tau - \omega, \omega_t)E_{\text{pump}}(\omega)E_{\text{pump}}(\omega_\tau - \omega)E_{\text{probe}}(\omega_t)\,. \tag{S5}$$

Note that the physical frequency of the nonlinear signal is $\omega = \omega_\tau + \omega_t$.

For the numerical result in the main text, we also included the heating effect by adding a phenomenological term:

$$\Delta E_{\text{heating}}(\omega_t, \omega_\tau) = c_h e^{-\omega_\tau^2/W_h^2}\Delta E(\omega_t, \omega_\tau) \tag{S6}$$

where $c_h = 1$ and $W_h = 1$ ($W_h = 0.7$) are fitting parameters for single-cycle (multi-cycle) THz pump. The extra Gaussian profile centering at $\omega_\tau = 0$ represents that fact that heating generates a non-oscillating decay of the change of reflectivity as the pump probe delay τ increases.

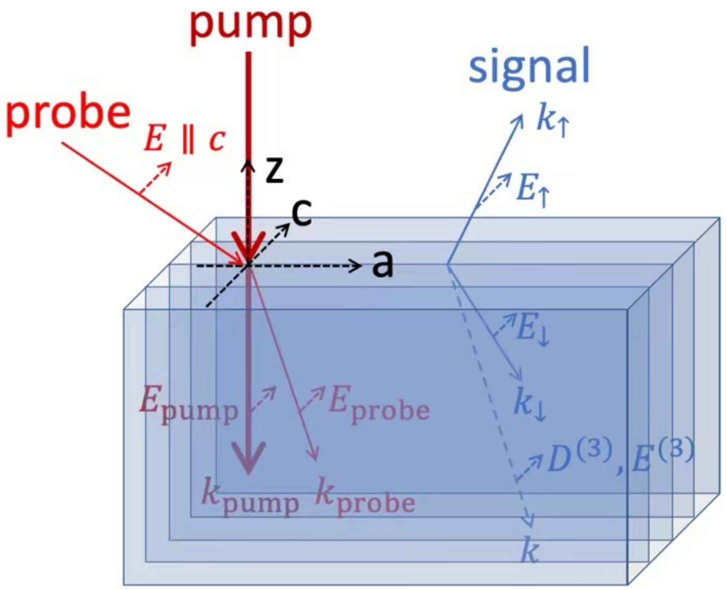

Figure S4: Set up of the measurement. All electric fields are parallel to the c-axis. Red arrows represent pump and probe EM waves and blue arrows are third order EM waves.

3.2 The emission coefficient

The electric fields from the pump and probe beams combine into the dynamical third order polarization:

$$D^{(3)}(\omega,\mathbf{k}) = \sum_{\omega_i,\mathbf{k}_i} \chi^{(3)}(\omega_1,\omega_2,\omega_3)E_{\text{pump}}(\omega_1,\mathbf{k}_1)E_{\text{pump}}(\omega_2,\mathbf{k}_2)E_{\text{probe}}(\omega_3,\mathbf{k}_3) \tag{S7}$$

where the constraint is $\omega_1+\omega_2+\omega_3=\omega$ and $\mathbf{k}_1+\mathbf{k}_2+\mathbf{k}_3=\mathbf{k}$.

The dynamical polarization at $(\omega,\mathbf{k})=(\omega,q,k_z)$ emits EM waves at three possible momenta, as shown by the blue arrows in Fig. S4. One is the response field at $(\omega,\mathbf{k})$ whose electric field component is denoted by $E^{(3)}$. The other two are $E_\downarrow$ at $(\omega,\mathbf{k}_\downarrow)=(\omega,q,k_{\downarrow z})=(\omega,q,-\sqrt{\epsilon_c k_0^2-q^2})$ inside the material, and $E_\uparrow$ at $(\omega,\mathbf{k}_\uparrow)=(\omega,q,k_{\uparrow z})=(\omega,q,\sqrt{k_0^2-q^2})$ above it. The latter is the signal read by the detector.

The response field $E^{(3)}$ is the linear response of the EM mode at $(\omega,\mathbf{k})$ to the perturbation $D^{(3)}$. The response can be determined from the Maxwell's equation:

$$\nabla\times\nabla\times\mathbf{E}^{(3)} = -\frac{\omega^2}{c^2}(\epsilon_c\mathbf{E}^{(3)}+\mathbf{D}^{(3)})\,,$$
$$E^{(3)} = \frac{k_0^2}{k^2-k_\downarrow^2}D^{(3)} \tag{S8}$$

where both $\mathbf{E}^{(3)}$ and $\mathbf{D}^{(3)}$ are along the c direction.

We now compute the emitted fields $E_\downarrow$ and $E_\uparrow$. Since their frequency-momenta are on shell, they exist as free solutions of the Maxwell's equation on each side of the interface even without external perturbation. However, in order for nonzero $E_\downarrow$ and $E_\uparrow$ to satisfy the boundary condition on the interface, an external source is necessary. In this case, the source is $\mathbf{E}^{(3)}$. The irreducible boundary condition is the continuity of magnetic field $\mathbf{B}=\frac{c}{i\omega}\nabla\times\mathbf{E}$ along a axis and electric field along c axis, which may be written as

$$\begin{cases} k_{\uparrow z}E_\uparrow = k_{\downarrow z}E_\downarrow + k_z E^{(3)}\,, \\ E_\uparrow = E_\downarrow + E^{(3)}\,. \end{cases} \tag{S9}$$

Eq. (S9) is solved to obtain

$$E_\uparrow = \frac{k_{\downarrow z}-k_z}{k_{\downarrow z}-k_{\uparrow z}}E^{(3)} = \frac{k_0^2}{(k_{\downarrow z}-k_{\uparrow z})(k_{\downarrow z}+k_z)}D^{(3)} \equiv F(\omega,\mathbf{k})D^{(3)} \tag{S10}$$

where $F(\omega,\mathbf{k})$ may be interpreted as the emission coefficient of the third order polarization into free space.

One may check the limit of pure heating effect where the pump uniformly heats up the material so that $\mathbf{k}=\mathbf{k}_\downarrow$ and ω is the same as the probing frequency. The emission coefficient Eq. (S10) reduces to $F\cdot T(\omega)\propto\partial_T R(\omega,q)$, i.e., the third order reflected field is just the probe field multiplied by the change of linear reflectivity due to heating.

The above formula can be readily applied to layered superconductors where the probing surface is the a-c surface, the incident plane is the a-b plane, and the pump/probe beam is TE polarized, same as the experimental configuration shown in Fig. S4.

3.3 Linear and nonlinear susceptibilities along the c-axis of layered superconductors

We assume the charge tunneling between the superconducting layers are weak so that it two adjacent layers are Josephson coupled. Considering only the electrodynamics along the c-axis, the effective Lagrangian for

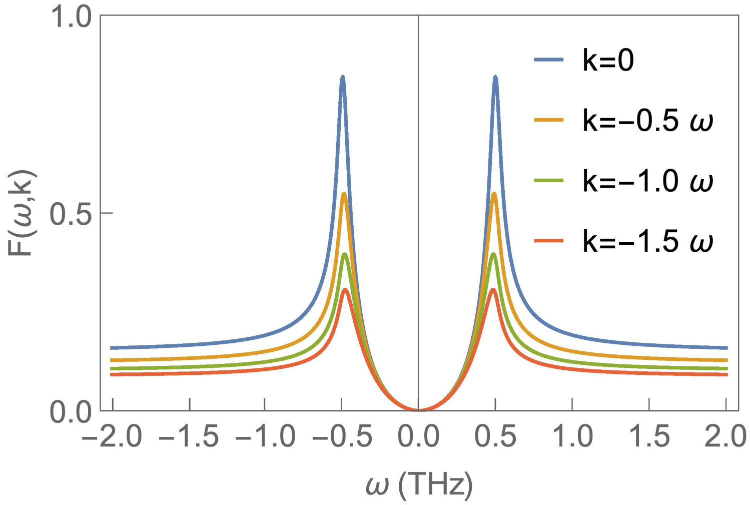

Figure S5: The absolution value of the emission coefficient Abs$[F(\omega, \mathbf{k})]$ from Eq. (S10) plotted as a function of ω and for $q = 0$ (zero incident angle for both the pump and probe) and several assumed k. For the ϵ_c in Eq. (S3), we used the experimentally measured values: $\omega_{\mathrm{JPR}} = 0.5\,\mathrm{THz}$ and $\epsilon_{c\infty} = 4.5$, and assumed a small scattering rate $\gamma = 0.06\,\mathrm{THz}$ to roughly represent the nonzero Im$[\epsilon_c]$ due to interband transitions and contributions from thermally excited quasiparticles. The peaks around $\omega = \pm 0.5\,\mathrm{THz}$ arise because $k_{\downarrow z} = -\sqrt{\epsilon_c} k_0$ vanishes at the Josephson plasma frequency where $\epsilon_c = 0$. In the $q = 0$ limit, the width of the peaks are controlled by Im$[\epsilon_c(\omega_{\mathrm{JPR}})]$, and could be very narrow. Note that in general, k is the sum of two pump and one probe wave vectors, which is a function of all three constitute frequencies and the incident angle. This plot is just a simplified version of F in order to see the potential peak at $\omega_\tau + \omega_t = \pm 0.5\,\mathrm{THz}$ in the summation in Eq. (S5).

such a system is a chain of Josephson coupled superconducting islands along z-direction [7]:

$$L = -\sum_n \left[\frac{1}{2}(\partial_t\theta_n + \phi_n)^2 + E_c \cos\left(\theta_{n+1} - \theta_n - \int_n^{n+1} A(z)dz\right)\right] \tag{S11}$$

where A is the vector potential along c-axis, ϕ is the scalar potential. For simplicity of notations, we have set the cooper pair charge $2e = 1$, the speed of light c, and the Planck constant $\hbar$ to unity. Now we compute the current response to a uniform electric field along c-axis using the gauge $\phi = 0, -\frac{1}{c}\partial_t A = E(t)$. The equation of motion for the phase θ implied by Eq. (S11) says the phase does not respond to uniform A because there is no charge accumulation, i.e., $\theta_n = 0$. Therefore, the current along z is [2, 1]

$$J(t) = \partial_A L = -J_c \sin\left(A(t)d\right) \tag{S12}$$

where d is the interlayer distance and $J_c = E_c d$ is the Josephson critical current. At linear order in A, Eq. (S12) gives the linear optical conductivity:

$$\sigma(\omega) = \frac{iD/\pi}{\omega}, \quad D = \pi J_c d(2e)/\hbar = \omega_{\mathrm{JPR}}^2/4 \tag{S13}$$

where we have restored $\hbar$ and the cooper pair charge $2e$. Expanding to third order in A, we obtain the third order nonlinear optical conductivity and the equivalent nonlinear susceptibility:

$$\begin{aligned}\sigma^{(3)}(\omega_1,\omega_2,\omega_3) &= \frac{iD^{(3)}}{\omega_1\omega_2\omega_3}, \quad D^{(3)} = \frac{1}{3!}J_c d^3(2e)^3/\hbar^3\,, \\ \chi^{(3)}(\omega_1,\omega_2,\omega_3) &= -\frac{D^{(3)}}{(\omega_1+\omega_2+\omega_3)\omega_1\omega_2\omega_3}\,. \end{aligned} \tag{S14}$$

Note that nonzero nonlinear conductivity implies broken Galilean invariance. In general, the underlying lattice means the electron band structure is periodic in momentum space, and must deviate from the Galilean invariant ($\varepsilon \propto p^2$) limit. In the current case, the local Josephson tunneling plays a similar role as the periodic band structure. In all these examples, the frequency dependence of $\sigma^{(3)}$ share the same universal 'hydrodynamic' form [6].

3.3.1 The two-plasmon contribution

In addition to the tree level contribution $\chi^{(3)}$ to the third order response, there are also higher order terms such as the one loop contribution $\chi^{(3)}_{\mathrm{loop}}$ investigated by Gabriele et al. [3], which may also be called the 'two-plasmon' contribution or the 'parametric resonance' contribution. At low temperatures ($T \ll \omega_{\mathrm{JPR}}$), this contribution is a quantum effect of the model in Eq. (S11), meaning that it is proportional to quantum fluctuations of the Josephson plasmons. To quantitatively model our sample, one needs to go beyond the single mode approximation used in Ref. [3] and sum the contribution from all the hyperbolic Josephson plasmons (see Fig.1b of Ref. [3] and our Sec. 3.4.1), yielding a continuum instead of a single peak:

$$\chi^{(3)}_{\mathrm{loop}}(\omega) = \sum_{q,\omega'} \chi^{(3)2} \frac{1}{\omega'^2 - \omega_q^2} \frac{1}{(\omega' + 2\omega)^2 - \omega_{-q}^2}\,. \tag{S15}$$

The absorption part, $\mathrm{Im}[\chi^{(3)}_{\mathrm{loop}}(\omega)]$, follows the density of states of the bulk Josephson plasmons, which jumps from zero to a nonzero value at ω_{JPR} as ω increases (see Sec. 3.4.1). From Eq. (S19), at the frequency

$\omega = \omega_{\mathrm{JPR}}$, the parametric resonance contribution is smaller than the tree level result (Eq. (S14)) by a factor of

$$\frac{\mathrm{Im}[\chi^{(3)}_{\mathrm{loop}}]}{\chi^{(3)}} \sim \left(J_c d^3 (2e)^3 \frac{1}{\hbar^3}\right) \hbar \Lambda^3 \frac{\omega_{\mathrm{JPR}}}{\omega_p^2} \frac{1}{\omega_{\mathrm{JPR}}^2} \sim \frac{1}{\hbar} \omega_{\mathrm{JPR}}^2 d^2 e^2 \Lambda^3 \frac{\omega_{\mathrm{JPR}}}{\omega_p^2} \frac{1}{\omega_{\mathrm{JPR}}^2} \sim \frac{e^2}{\hbar d} \frac{\omega_{\mathrm{JPR}}}{\omega_p^2} \ll 1\,. \tag{S16}$$

In the last inequality, we have used the estimate that $\frac{e^2}{d} \sim 1\,\mathrm{eV} \sim \hbar\omega_p$.

We note that since there is a jump of $\mathrm{Im}[\chi^{(3)}_{\mathrm{loop}}(\omega)]$ at $\omega = \omega_{\mathrm{JPR}}$, from the Kramers-Kronig relation, there may be a logarithmic divergence of $\mathrm{Re}[\chi^{(3)}_{\mathrm{loop}}(\omega)]$ close to ω_{JPR}. This weak divergence may provide another mechanism for enhanced signal around ω_{JPR}, which warrants further investigation.

3.4 The Josephson plasmons

3.4.1 Bulk plasmons

The longitudinal Josephson plasmons are equivalently characterized by the dielectric function tensor which includes the c-axis one ϵ_c in Eq. (S3) and that along the ab plane. A simple model for the latter is

$$\epsilon_{ab}(\omega) = \epsilon_{ab\infty}\left(1 - \frac{\omega_p^2}{\omega(\omega + i\gamma)}\right)\,. \tag{S17}$$

Solving the Maxwell's equation for the electromagnetic (EM) wave with the dielectric being Eq. (S17) and Eq. (S3), one obtains the dispersion of the bulk Josephson plasmons

$$\frac{q_a^2 + q_b^2}{\epsilon_c(\omega)} + \frac{q_c^2}{\epsilon_{ab}(\omega)} = \frac{\omega^2}{c^2} \tag{S18}$$

which exist within the frequency regime $\omega_{\mathrm{JPR}} < \omega < \omega_p$ such that $\epsilon_c \epsilon_{ab} < 0$, i.e., the 'Reststrahlen' band. These are called *hyperbolic Josephson plasmons* [7] because at each frequency in the Reststrahlen band, the momenta of the modes span a hyperbola in the momentum space. In the 'near field' limit, meaning when the momentum q and frequency ω of the modes satisfy $cq \gg \omega$, the dispersion simplifies to $\epsilon_{ab}(\omega)(q_a^2 + q_b^2) + \epsilon_c(\omega) q_c^2 = 0$. which approximates the hyperbolas by cones. Note that Eq. (S3) involves a local approximation for the dielectric function, which is good for $q \ll \Lambda \sim 1/d$, the lattice scale [7, 3].

To compute the density of the states (DOS) of the bulk Josephson plasmons, it is enough to use the near field approximation since the momentum cutoff for the hyperbolic dispersion is the lattice scale, much larger than ω/c. It reads:

$$g(\omega) = \frac{4\pi}{3(2\pi)^3}\Lambda^3 \frac{2\pi \sin\theta d\theta}{4\pi d\omega} = \frac{\Lambda^3}{3(2\pi)^2}(\sin\theta)\partial_\omega \theta \tag{S19}$$

where $\theta(\omega) = \mathrm{ArcTan}\left[\sqrt{\left|\frac{\epsilon_c(\omega)}{\epsilon_{ab}(\omega)}\right|}\right]$ is half of the angle of the cone surface. Close to the lower boundary of the Reststrahlen band ($\omega = \omega_{\mathrm{JPR}}$), the DOS approaches a constant: $g(\omega) \sim \Lambda^3 \omega_{\mathrm{JPR}}/\omega_p^2$. Therefore, there is no peak at $\omega = \omega_{\mathrm{JPR}}$.

3.4.2 Surface plasmons

We focus on the case where the interface is the a-c plane, as in Fig. S4. In the Reststrahlen band $\omega_{\rm JPR} < \omega < \omega_p$, the surface mode satisfies [8]

$$\epsilon_{ab}\left[\epsilon_{ab}\left(\frac{q_a}{q}\right)^2 + \epsilon_c\left(\frac{q_c}{q}\right)^2\right] = 1 \tag{S20}$$

where $q = \sqrt{q_a^2 + q_c^2}$. Therefore, for fixed frequency, the momenta of the surface modes form a cone on the $q_a - q_c$ plane, meaning that they are *hyperbolic surface plasmons*. The surface modes satisfying Eq. (S20) exist in the frequency range $\omega_s < \omega < \omega_{\rm JPR}$ where ω_s satisfies $\epsilon_{ab}(\omega_s)\epsilon_c(\omega_s) = 1$. Because $\omega_p \gg \omega_{\rm JPR}$, one has the approximate solution $\omega_s = \omega_{\rm JPR}\left(1 - \frac{\omega_{\rm JPR}^2}{2\epsilon_{ab\infty}\epsilon_{c\infty}\omega_p^2}\right)$ which is below but very close to $\omega_{\rm JPR}$.

References

[1] Vinay Ambegaokar and Alexis Baratoff. Tunneling between superconductors. *Phys. Rev. Lett.*, 10:486–489, Jun 1963.

[2] D. N. Basov and T. Timusk. Electrodynamics of high-T_c superconductors. *Rev. Mod. Phys.*, 77:721–779, Aug 2005.

[3] Francesco Gabriele, Mattia Udina, and Lara Benfatto. Non-linear terahertz driving of plasma waves in layered cuprates. *Nature communications*, 12(1):1–8, 2021.

[4] H. Hirori, A. Doi, F. Blanchard, and K. Tanaka. Single-cycle terahertz pulses with amplitudes exceeding 1 mv/cm generated by optical rectification in linbo3. *Applied Physics Letters*, 98(9):091106, 2011.

[5] Jian Lu, Xian Li, Harold Y. Hwang, Benjamin K. Ofori-Okai, Takayuki Kurihara, Tohru Suemoto, and Keith A. Nelson. Coherent two-dimensional terahertz magnetic resonance spectroscopy of collective spin waves. *Phys. Rev. Lett.*, 118:207204, May 2017.

[6] Zhiyuan Sun, D. N. Basov, and M. M. Fogler. Third-order optical conductivity of an electron fluid. *Phys. Rev. B*, 97:075432, Feb 2018.

[7] Zhiyuan Sun, M. M. Fogler, D. N. Basov, and Andrew J. Millis. Collective modes and terahertz near-field response of superconductors. *Phys. Rev. Res.*, 2:023413, Jun 2020.

[8] Zhiyuan Sun, Á. Gutiérrez-Rubio, D. N. Basov, and M. M. Fogler. Hamiltonian optics of hyperbolic polaritons in nanogranules. *Nano Letters*, 15(7):4455–4460, 07 2015.

[9] S. J. Zhang, Z. X. Wang, H. Xiang, X. Yao, Q. M. Liu, L. Y. Shi, T. Lin, T. Dong, D. Wu, and N. L. Wang. Photoinduced nonequilibrium response in underdoped $yba_2cu_3o_{6+x}$ probed by time-resolved terahertz spectroscopy. *Phys. Rev. X*, 10:011056, Mar 2020.
